# Results from the Balance Rehabilitation Unit in Benign Paroxysmal Positional Vertigo

**DOI:** 10.1590/S1808-86942010000500015

**Published:** 2015-10-22

**Authors:** Cristiane Akemi Kasse, Graziela Gaspar Santana, Renata Coelho Scharlach, Juliana Maria Gazzola, Fátima Cristina Barreiro Branco, Flávia Doná

**Affiliations:** 1 ENT, MSc and PhD - Federal University of São Paulo; Professor of the Body Balance and Social Inclusion Program of Uniban - Brazil; 2 Speech and hearing therapist; MSc - Body Balance and Social Inclusion Program of Uniban - Brazil; 3 Speech and Hearing Therapist. PhD - Human Communications Disorders; Professor - MSc Program in Body Balance and Social Inclusion Program of Uniban - Brazil; 4 Physical therapist and MSc in Sciences, Professor at the Body Balance and Social Inclusion Program of Uniban - Brazil; 5 Speech and Hearing Therapist and PhD in Psychology; Professor of the Body Balance Rehabilitation and social inclusion Graduate Program - UNIBAN - Brazil; 6 Physical Therapist and PhD in Sciences, Professor of the Body Balance Rehabilitation and social inclusion Graduate Program - UNIBAN - Brazil

**Keywords:** aged, posture, vertigo.

## Abstract

**Abstract:**

Posturography is a useful new tool to study the influence of vestibular diseases on balance.

**Aim:**

to compare the results from the Balance Rehabilitation Unit (BRU) static posturography in elderly patients with Benign Paroxysmal Positional Vertigo (BPPV), before and after Epley's maneuver.

**Materials and Methods:**

a prospective study of 20 elderly patients with a diagnosis of BPPV. The patients underwent static posturography and the limit of stability (LE) and ellipse area were measured. We also applied the Dizziness Handicap Inventory (DHI) questionnaire to study treatment effectiveness.

**Results:**

80% were females, with a mean age of 68.15 years. After the maneuver, the LE increased significantly (p=0.001). The elliptical area of somatosensory, visual and vestibular conflicts (2,7,8,9 situations) in BRU and the DHI scores decreased significantly (p<0.05) after treatment.

**Conclusion:**

the study suggests that elderly patients with BPPV may present static postural control impairment and that the maneuver is effective for the remission of symptoms, to increase in the stability and improvement in postural control in situations of visual, somatosensory and vestibular conflicts.

## INTRODUCTION

Benign Paroxysmal Positional Vertigo (BPPV) is characterized by the presence of rotational dizziness episodes upon change in head position (as lying down towards one side, standing up and/or looking upwards. Moreover, in between spell intervals, the patient can report body instability, other types of dizziness, gait deviation and functional disability.^1^

BPPV is considered the most common of the positional vertigo disorders and it is the main cause of vertigo among adults and senior citizens.^1^, ^2^, ^3^, ^4^ its pathophysiology is associated to the shifting of utricular statocone debris in a disorganized fashion towards the semicircular canal (SCC). If these two debris remain floating on the endolymph, along the SCC involved, cause an abnormal shifting of the cupula upon head movements in the affected SCC plane, we call this process canalolithiasis. Nonetheless, if these two debris adhere to the SCC dome, its density changes, causing an inadequate shifting upon head movement. This process is called cupulolitíase.^2^,^3^

The assessment of which SCC is involved is done by means of Dix & Hallpike and Brandt & Daroff diagnostic tests.^2^,^4^ The posterior SCC involvement by canalolithiasis is responsible for more than 95% of the cases of BPPV and in this case, especially when it occurs in the superior SCC, the treatment is carried out through the Epley or Semont statocones repositioning maneuvers.^5^, ^6^, ^7^ Idiopathic etiologies correspond to the majority of cases of the disease3. The other etiologies are: sedentary life, cranial trauma, inadequate diets, hyperlipidemia, hypo or hyperglycemia, hyperinsulinemia, vascular changes, Ménière's disease and vestibular neuritis.^1^,^3^

Body balance maintenance happens because of the information received from three sensorial systems, namely: visual, vestibular and proprioceptive, and it depends on the action of vestibulo-ocular, vestibular-spinal and optokinetic reflexes.^8^ It is very likely that the BPPV affect both the vestibulo-ocular and the vestibular-spinal reflexes.^9^

In recent years there are some virtual reality systems and force platforms aimed at improving body balance and balance system rehabilitation.^10^ The Balance Rehabilitation Unit (BRU^TM^) measures balance disorders by means of measuring the shifting areas from the pressure center, translated into Portuguese from body center of pressure, which acronym used in the literature is COP and, for such reason, we used it in this paper, ten sensorial condition associated to visual, vestibular and somatosensory reflexes.^11^

In order to analyze the efficacy of the therapeutic maneuvers and the clinical impact of BPPV on the patients, the studies are based, mainly, on the complaints, the lack of nystagmus and in the results from the handicap questionnaire scores, such as the Dizziness Handicap Inventory.^12^,^13^ There are few studies on body balance characteristics of patients with BPPV, especially the posturography values, and none of them specifically investigates the group of elderly patients with BPPV by means of BRU^TM^ static posturography.

This study aimed at comparing the results from the static posturography by means of the Balance Rehabilitation Unit (BRU^TM^), in elderly patients with benign paroxysmal positional vertigo before and after the Epley's statocone repositioning maneuver.

## PATIENTS AND METHODS

The present study was submitted to the Ethical Rules and Regulations Standards of the University, which approved it according to Protocol #2009-318.

This is a clinical-prospective study carried out in elderly patients diagnosed with BPPV, who were evaluated by an ENT physician from January to July of 2009. The patients agreed and signed the free and informed consent form. We included 20 patients with age above 60 years, males and females; without the use of anti-vertigo or psychotropic medication; who did not previously undergo vestibular rehabilitation or maneuvers.

The exclusion criteria were patients unable to understand simple verbal commands; or remain in orthostatic position, without help during the posturography; with advanced degenerative diseases; moderate to severe neck spinal disorders; people in clutches, canes or walks; people with severe auditory and/or visual involvement; patients with other concurrent vestibular diseases.

The tests ran before and after the Epley's repositioning maneuver were: for BPPV diagnostic confirmation; the diagnostic tests of Brandt-Daroff and Dix-Hallpike which were considered positive in the presence of nystagmus or vertigo^4^,^15^; the ten BRU^TM^ static posturography situations, the DHI questionnaire in its Brazilian version.^16^,^17^

The BRU^TM^ posturography module provides information on the center of pressure position (COP) of the patient by means of quantitative indicators: stability limit area (SLA) and elliptical area, in ten sensorial conflict situations.^10^,^11^

COP is the point of application of the resulting vertical forces acting on the support surface and it represents a collective result from the postural control system and the gravitational force.^18^ In order to measure SLA and the elliptical areas of the aforementioned situations, the patient was placed in the standing up position, with the arms extended along the body on the balance platform. The posturography technique and parameters followed the method described by Gazzola et al. (2009).^10^ The sensorial conflict situations are depicted on Chart 1.

In order to treat BPPV, the statocone repositioning maneuver chosen was the one described by Epley and later modified, without the use of the bone vibrator, without postural restrictions because of its good effeciveness.^5^,^6^,^19^,^20^ In each case we considered the canal involved and the nystagmus duration, and this was weekly repeated until symptom resolution. Having symptom remission confirmed, the patients were submitted to a posturography evaluation and application of the DHI questionnaire. The patients in whom dizziness not associated with characteristic BPPV symptoms persisted after the therapeutic maneuver were submitted to the computerized vector-electronystagmography and were taken off the study.

The Wilcoxon Signed Ranks Test was used in order to compare the DHI, SLA and elliptical area results in the ten pre and post Epley maneuver situations. In order to calculate the values we used the SPSS 10.0 for Windows (Statistical Package For Social Sciences, version 10.0, 1999) software and the significance level used was lower than 5%.

## RESULTS

Of the 20 patients with a diagnostic suspicion of BPPV, sixteen (80%) were women, with a mean age of 68.15 years and standard deviation of 6.06 years.

Positional nystagmus study indicated unilateral involvement of the posterior semicircular canal in 14 (70%) patients, and eight of them had it in the right canal. Five (25%) patients had bilateral involvement of the posterior semicircular canal and one (5%) had unilateral involvement of the left anterior semicircular canal.

All the patients had BPPV of the canalolithiasis type, 50% of the cases resolved it with only one maneuver, 35% after two maneuvers and the remaining required three maneuvers.

After Epley's maneuver, there were significant differences in the total DHI score and subscales (p< 0.0001), followed by the lack of symptoms and nystagmus in the diagnostic test (Table 1).

**Chart 1:** Types of stimuli received by the virtual reality goggles during 60 seconds and surface types with the individual in the orthostatic position in 10 sensorial situations, in the Balance Rehabilitation Unit (BRU ^T^).

Situations	Types of Stimuli and Surfaces
1	FS, no stimuli, OE
2	No stimuli, FS, CE
3	No stimuli, foam surface, CE
4	Saccadic, FS, OE
5	Optokinetic, bars (to the right) FS, OE
6	Optokinetic, bars (to the left), FS, EO
7	Optokinetic, bars (downwards): FS, OE
8	Optokinetic, bars (upwards): FS, OE
9	Visual-vestibular interaction, circular, bars (horizontal direction), FS, OE
10	Vestibular-visual interaction, circular, bars (vertical direction) FS, OE

Legend:

FS - firm surface

OE - open eyes

CE - closed eyes

**Figure 1:**
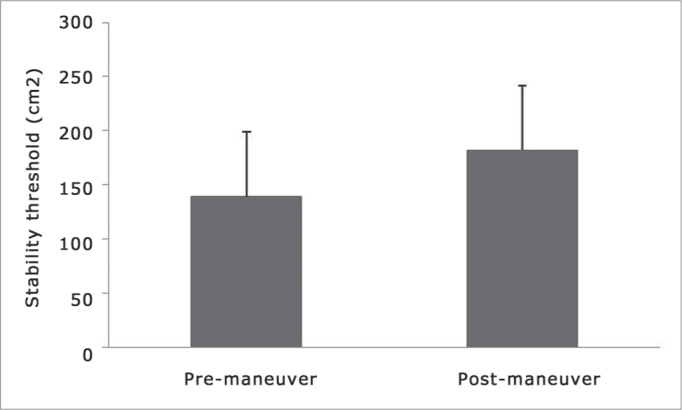
Stability Limit Area (SLA) before and after Epley's maneuver. *Wilcoxon Signed Ranks Test (p=0,001).

Insofar as the posturography results go, SLA showed a statistically significant difference (p=0.001) when compared to pre (139.05 ± 59.96 cm^2^) and post (181.85 ± 45.76 cm2) Epley' maneuver (Figure 1).

Table 1, Table 2 depicts the descriptive values and the comparative analysis of the elliptical area of the ten BRU^TM^ situations, before and after the Epley's maneuver. There were statistically significant differences before and after the maneuver in situations 2, 7, 8 and 9.

**Table 1:** Mean and standard-deviation values and descriptive level obtained in relation to the physical, functional, emotional aspects and total Dizziness Handicap Inventory score (Brazilian version) in patients with benign paroxysmal positional vertigo before and after the Epley's maneuver.

Dizziness Handicap Inventory	Pre Epley's Maneuver	Post Epley's Maneuver	p-value^*^ (Wilcoxon test)
	Mean (Standard Deviation)	Mean (Standard Deviation)	
Physical	2,43 (1,05)	0,10 (0,44)	< 0,0001
Functional	1,50 (0,96)	0,77 (0,26)	< 0,0001
Emotional	0,76 (0,87)	0,02 (0,09)	< 0,0001
Total	37,60 (21,12)	1,60 (6,27)	< 0,0001

^*^Wilcoxon Signed Ranks Test

**Table 2:** Descriptive values and comparative analysis of the COP area under the 10 BRUT COP situations before and after the Epley's maneuver in elderly patients with BPPV.

BRU^T^ situations	Epley' maneuver	Mean COP values (Standard Deviation) (cm^2^)	COP values variation (cm^2^)	Median COP values (cm^2^)	p-value*
	Pre	3,19 (2,28)	0,45 - 7,97	2,71	
FS/Open eyes/No Stimulus					0,401
	Post	3,02 (3,77)	0,60 - 17,46	1,68	
	Pre	4,46 (3,9)	0,37 - 15,26	2,60	
FS/Open eyes					0,003
	Post	2,34 (3,21)	0,50 - 15,13	1,20	
	Pre	12,19 (13,27)	2,43 - 62,56	7,90	
Foam/Eyes Shut					0,062
	Post	7,94 (3,87)	1,53 - 16,76	6,90	
	Pre	1,94 (1,05)	0,72 - 4,88	1,75	
FS/Saccadic					0,230
	Post	1,73 (0,89)	0,53 - 3,22	1,44	
	Pre	2,54 (2,03)	0,54 - 8,66	1,77	
FS/Bars/Optokinetic to the right					0,083
	Post	2,13 (2,83)	0,36 - 13,56	1,35	
	Pre	3,24 (3,6)	0,55 - 16,51	2,01	
FS/Bars/Optokinetic to the left					0,079
	Post	2,38 (2,81)	0,36 - 13,62	1,67	
	Pre	3,26 (3,49)	0,75 - 16,47	2,08	
FS/Bars/Optokinetic downwards					0,018
	Post	2,16 (3,5)	0,24 - 16,84	1,49	
	Pre	2,58 (2,14)	0,68 - 8,42	1,86	
FS/Bar/Optokinetic upwards					0,037
	Post	1,88 (1,65)	0,40 - 8,01	1,32	
FS/Bars/Visual-Vestibular Interaction /	Pre	4,31 (2,17)	1,58 - 10,05	3,96	0,040
Horizontal Direction	Post	3,58 (2,15)	1,44 - 10,83	3,21	
FS/Bars/Visual-Vestibular Interaction /	Pre	3,76 (1,78)	1,30 - 8,54	3,53	0,247
Vertical Direction	Post	3,31 (2,16)	1,12 - 9,49	2,91	

FS: Firm Surface.

Significance Level at = 0.05, FS: Firm Surface,

* Wilcoxon Signed Ranks Test

**Figure 2:**
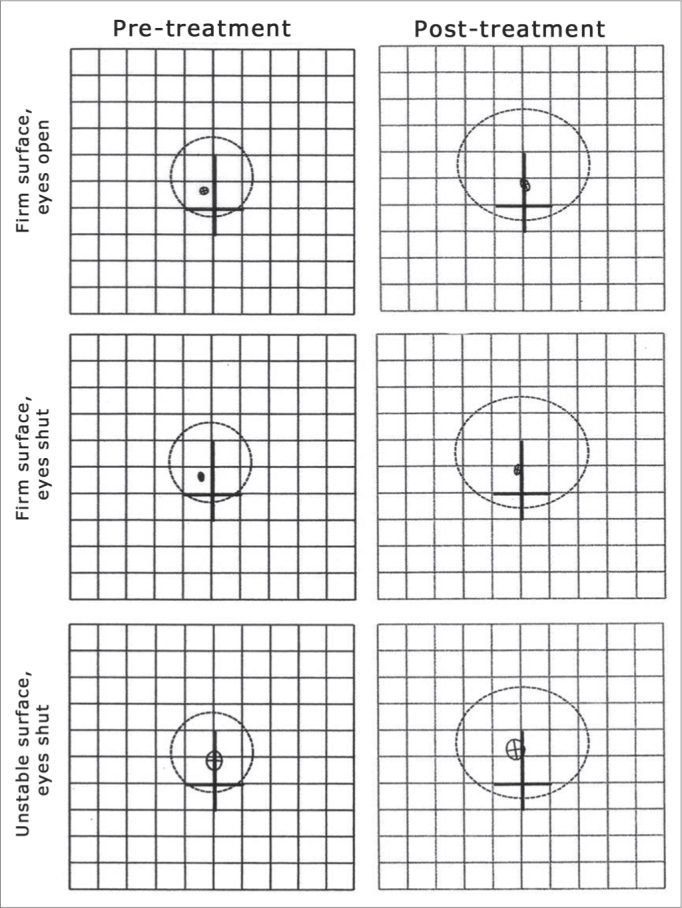
Balance Rehabilitation Unit posturography, in the elderly with BPPV, before and after Epley's maneuver on the situations of firm surface with eyes open and closed and firm surface with eyes shut. The greater circle is represented by SLA and the smaller one by COP. After the maneuver, SLA increased and COP reduced.

Figure 2 shows situations 1, 2 and 3 of the BRU^TM^ before and after Epley's maneuver in one of the patients in the study: situation 1: firm surface and eyes open; situation 2: firm surface and eyes shut; and situation 3: unstable surface and eyes shut.

## DISCUSSION

The present investigation showed that the modified Epley's maneuver in elderly patients with BPPV caused dizziness and nystagmus remission, it reduced the Dizziness Handicap Inventory (DHI) score and improved postural control upon BRU^TM^ posturography. BPPV is the most common cause of vertigo in the population above 50 years of age and it can be easily diagnosed and treated by means of maneuvers used to reposition statocone particles, ratified by the present study and by the literature.^9^,^14^,^21^,^22^

Patients who suffer from BPPV usually report that the dizziness starts when they are laying down, change position and tilt their trunk forward or look upwards.^3^,^5^,^15^ These symptoms are triggered by the abnormal movement of the endolymph, induced by statocones in the lumen of the canals or when they adhere to the ampoule dome, making the semicircular canal into an organ that is sensitive to gravitational movement, causing this feeling of unbalance and postural instability^3^,^15^.

Body balance is a complex process which depends solely on the integration of the visual, vestibular an somatosensory systems, central coordination and muscular adjustment, especially the tonus muscles.^23^ The postural muscles are activated by reflex mechanisms and active control of body movements in order to maintain the center of gravity within stability limits.^23^,^24^ BRU^TM^ static posturography enables the evaluation of body balance in different situations of sensory conflicts by means of a computerized posturography module, with visual stimuli projections in virtual reality goggles.^10^,^11^

The commonly used posturography measure is the COP, which is the point at which the resulting force from the vertical forces act on a support surface.^18^ We did not observe pertaining studies in the literature on the assessment of body balance in the elderly nor in adults with BPPV before and after Epley's maneuver using the static BRUTM posturography.

The parameters evaluated by means of the BRUTM posturography were: the Stability Limit Area (SLA) and the COP shifting area in ten sensorial situations. We also noticed a significant increase in SLA after Epley's maneuver, which suggests a greater body stability and better control over the ankle reactive strategy.

Static posturography assesses the patient's willingness to tilt anteroposteriorly and side to side, withoutchanging his/her support base or take a step or use the hip-reactive strategy. Elderly patients with vestibular dysfunction can have SLA reduction and that of the ankle and hip reactive strategies which can cause functional disability and the risk of falls.^25^, ^26^, ^27^

It was not possible to notice significant COP changes on situation 1 (stable surface and eyes open), in other words, somatosensory and visual information present. In our study, this finding corroborates those from other authors, in which patients with vestibular disorders use, especially, visual and/or proprioceptive clues in order to maintain body balance^23^,^27^.

This study has also revealed that the elderly assessed on situation 2 (stable surface and eyes shut), in other words, with visual information, had a reduction in COP (p=0.003) area after the maneuver, suggestive that the treatment enabled a better body balance maintenance on the still vertical posture.

Upon situation 3 (unstable surface and eyes shut), in other words, without visual clues and with the inaccurate somatosensory information, the elderly with BPPV presented, after the maneuver, a tendency towards reducing the COP area (p=0.062), indicating a greater postural control after treatment, with the accurate information coming from the vestibular system and greater integration of the somatosensory information (visual, vestibular and somatosensory).

According to Horak (1990), in a comparative study between normal individuals and those with vestibular function loss; and have noticed that when vestibular and somatosensory information are inaccurate or impaired, the body tilt of the patients with vestibular affection was significantly higher, bringing about body balance loss at the time when the vestibular system was acting alone.^28^

On situations 4, 5 and 6, the visual stimuli were projected on a virtual reality goggle, without changes seen on somatosensory information. The patients did not present statistical differences after the Epley's maneuver. Such situations assess the effects of visual conflicts by means of saccadic movements (situation 4). These situations assess the effects of visual conflicts by means of saccadic movements (situation 4) and horizontal optokinetic stimulation (situations 5 and 6) in postural control.^11^

Nonetheless, under situations 7 and 8, with vertical optokinetic stimulation, there were significant reductions in COP values, on patients after the maneuver, indicating that the vertical optokinetic stimulus can be more stimulating in relation to the horizontal bars.

Under situations 9 and 10, we assessed the visualvestibular integration by means of optokinetic stimuli associated with head movements, in the horizontal and vertical situations, respectively. Under situation 9, the results proved that after the Epley's maneuver there was a significant reduction in COP values (p=0.040). Nonetheless, on situation 10, there was no significant difference (p=0.25). These results lead us to infer that horizontal head movements, associated with optokinetic stimulation can be more stimulating or cause visual conflicts in relation to the head flexo-extension movement, associated to the optokinetic stimuli. Nonetheless, future investigations are needed to clarify these findings.

Giacomini et al. (2002) assessed the oscillation velocity by means of static posturography, however in BPPV adults, comparing them to the control group, then they perceived that even after the maneuver, the patients presented oscillation changes in the sagittal plane. The Epley's maneuver was effective for an improvement in the frontal plane oscillation, very likely associated with canalicular dysfunction; however, after the maneuver there was no influence on the sagittal oscillation plane, persisting for 12 months after treatment.^29^ The authors associate this finding to a change in the vestibular-spinal reflex, of still unknown origin.^29^

Stambolieva and Angov (2005) also assessed body balance in adults with BPPV, before and after Epley's maneuver, and compared with healthy adults, using static posturography under the situations of open eyes and eyes shut.^9^ The authors noticed that even after the maneuver, the BPPV patients, although asymptomatic, had body balance changes, very likely because of the statocones which alter canal physiology, changing the sensitiveness of its receptors and causing permanent macula dysfunction.^9^

For the authors, the maneuvers did not treat the lesions caused by the migration of statocones in the vestibular system, it was efficient only to reduce visualvestibular conflict, which after eliminating symptoms and the nystagmus there would be compensatory and adaptative mechanisms from other sensorial systems for maintaining body balance in the still vertical posture.^9^ It is important to stress that the present study also showed that in some static BRU^TM^ situations there were no changes to the COP area after Epley's maneuver, which may or may not be associated to the theory proposed by Stambolieva and Angov (2005).^9^

In order to analyze the efficacy of the therapeutic maneuvers and their impact on the patients with BPPV, most of the studies described in the literature are based on patient complaint, on DHI and/or on the absence of nystagmus, matching our results^2^,^12^,^13^,^22^,^30^. The current study also showed that the BRUTM static posturography module can provide objective results in order to assess elderly patients with BPPV, with the involvement of vertical canals and it can be considered a valuable instrument for the documentation and monitoring of disease treatment which specifically affect these canals.

We must also stress that despite the small sample, there was no loss concerning result analyses concerning patients with concurrent BPPV and other vestibular affections or with somatosensory or visual or musculoskeletal changes which could act as biases, compromising the result and the conclusion of tests.

## CONCLUSION

Elderly patients with BPPV, of the canalolithiasis type showed an increase in the stability threshold and improvements in the postural control in visual and somatosensory conflict situations, seen upon static Balance Rehabilitation Unit posturography, after the Epley's statocone repositioning maneuver, concurrent to clinical and quality of life improvements.
